# Electron Density and Effective Atomic Number as Quantitative Biomarkers for Differentiating Malignant Brain Tumors: An Exploratory Study with Machine Learning

**DOI:** 10.3390/tomography11110120

**Published:** 2025-10-29

**Authors:** Tsubasa Nakano, Daisuke Hirahara, Tomohito Hasegawa, Kiyohisa Kamimura, Masanori Nakajo, Junki Kamizono, Koji Takumi, Masatoyo Nakajo, Fumitaka Ejima, Ryota Nakanosono, Ryoji Yamagishi, Fumiko Kanzaki, Hiroki Muraoka, Nayuta Higa, Hajime Yonezawa, Ikumi Kitazono, Jihun Kwon, Gregor Pahn, Eran Langzam, Ko Higuchi, Takashi Yoshiura

**Affiliations:** 1 Department of Radiology, Kagoshima University Graduate School of Medical and Dental Sciences, 8-35-1 Sakuragaoka, Kagoshima 890-8544, Japan; tnakano0722@gmail.com (T.N.); ffieldai@gmail.com (D.H.); jinhasejinjin@yahoo.co.jp (T.H.); masanori.nakajo.0711@gmail.com (M.N.); operation.desert-fox.xfa-27@hotmail.co.jp (J.K.); takumi@m2.kufm.kagoshima-u.ac.jp (K.T.); toyo.nakajo@dolphin.ocn.ne.jp (M.N.); ejm.1107@gmail.com (F.E.); igkb2067@yahoo.co.jp (R.N.); apple2015ryoji2015@gmail.com (R.Y.); kanzaki.klarin.fumiko@gmail.com (F.K.); k3049025@kadai.jp (H.M.); 2 Department of Management Planning Division, Harada Academy, 2-54-4 Higashitaniyama, Kagoshima 890-0113, Japan; 3 Department of Advanced Radiological Imaging, Kagoshima University Graduate School of Medical and Dental Sciences, 8-35-1 Sakuragaoka, Kagoshima 890-8544, Japan; kiyohisa@m2.kufm.kagoshima-u.ac.jp; 4 Department of Neurosurgery, Kagoshima University Graduate School of Medical and Dental Sciences, 8-35-1 Sakuragaoka, Kagoshima 890-8544, Japan; nayuhiga@gmail.com (N.H.); hajime@m3.kufm.kagoshima-u.ac.jp (H.Y.); 5 Department of Pathology, Kagoshima University Graduate School of Medical and Dental Sciences, 8-35-1 Sakuragaoka, Kagoshima 890-8544, Japan; iky193@m2.kufm.kagoshima-u.ac.jp; 6 Philips Japan Ltd., 1-3-1 Azabudai, Minatoku, Tokyo 106-0041, Japan; jihun.kwon@philips.com (J.K.); koh.higuchi@philips.com (K.H.); 7 Philips GmbH Market DACH, Rontgenstr. 22, 22335 Hamburg, Germany; gregor.pahn@philips.com; 8 Philips Healthcare, Nahum Het St 16, Halfa 3100202, Israel; eran.langzam@philips.com

**Keywords:** dual-energy CT, electron density, effective atomic number, brain metastasis, glioblastoma, primary central nervous system lymphoma

## Abstract

**Objectives:** The potential use of electron density (ED) and effective atomic number (Zeff) derived from dual-energy computed tomography (DECT) as novel quantitative imaging biomarkers for differentiating malignant brain tumors was investigated. **Methods:** Data pertaining to 136 patients with a pathological diagnosis of brain metastasis (BM), glioblastoma, and primary central nervous system lymphoma (PCNSL) were retrospectively reviewed. The 10th percentile, mean and 90th percentile values of conventional 120-kVp CT value (CTconv), ED, Zeff, and relative apparent diffusion coefficient derived from diffusion-weighted magnetic resonance imaging (rADC: ADC of lesion divided by ADC of normal-appearing white matter) within the contrast-enhanced tumor region were compared across the three groups. Furthermore, machine learning (ML)-based diagnostic models were developed to maximize diagnostic performance for each tumor classification using the indices of DECT parameters and rADC. Machine learning models were developed using the AutoGluon-Tabular framework with rigorous patient-level data splitting into training (60%), validation (20%), and independent test sets (20%). **Results:** The 10th percentile of Zeff was significantly higher in glioblastomas than in BMs (*p* = 0.02), and it was the only index with a significant difference between BMs and glioblastomas. In the comparisons including PCNSLs, all indices of CTconv, Zeff, and rADC exhibited significant differences (*p* < 0.001–0.02). DECT-based ML models exhibited high area under the receiver operating characteristic curves (AUC) for all pairwise differentiations (BMs vs. Glioblastomas: AUC = 0.83; BMs vs. PCNSLs: AUC = 0.91; Glioblastomas vs. PCNSLs: AUC = 0.82). Combined models of DECT and rADC demonstrated excellent diagnostic performance between BMs and PCNSLs (AUC = 1) and between Glioblastomas and PCNSLs (AUC = 0.93). **Conclusion:** This study suggested the potential of DECT-derived ED and Zeff as novel quantitative imaging biomarkers for differentiating malignant brain tumors.

## Simple Summary

Electron density and effective atomic number derived from dual-energy CT have been used in various clinical applications, but their usefulness for brain tumor diagnosis remains unestablished. We investigated the potential utility of these novel CT parameters in differentiating brain metastases, glioblastomas, and primary central nervous system lymphomas. Effective atomic number differed across tumors, and the machine learning model using dual-energy CT parameters achieved high differentiating abilities. This study showed the potential of dual-energy CT-derived electron density and effective atomic number as novel quantitative imaging biomarkers for differentiating malignant brain tumors.

## 1. Introduction

Brain metastasis (BM), glioblastoma, and primary central nervous system lymphoma (PCNSL) are the most common malignant brain tumors in adults [1,2]. Imaging plays an extremely important role in the preoperative diagnosis; in particular, magnetic resonance imaging (MRI) is crucial for differentiating these tumors. Morphological features in conventional images can help differentiate these tumors, and some advanced MRI techniques, such as diffusion-weighted imaging (DWI), perfusion-weighted imaging, and MR spectroscopy, are useful [3,4]. The use of these multiparametric imaging modalities makes differentiation less challenging in such typical cases; however, in some atypical cases, these tumors can be very similar in imaging appearance, and preoperative differentiation can be challenging. Although computed tomography (CT) is also routinely performed a preoperative examination, the role of CT has only been supplemental in diagnosing brain tumors to date.

Both electron density (ED) and effective atomic number (Zeff) are parameters that influence CT values and have intrinsic values specific to each material [5]. Dual-energy CT (DECT) enabled the calculation of ED and Zeff and the CT value on a pixel-by-pixel basis [6]. Numerous studies have used ED and Zeff for tumor imaging and reported the usefulness of these novel imaging parameters for differentiating benign and malignant lesions or estimating histological subtypes [7,8,9,10,11]. However, few studies have assessed these images in brain tumors; therefore, the clinical value of these parameters remains unclear.

Simultaneously, machine learning (ML) for imaging diagnosis has been actively applied, and numerous studies on the differentiation of brain tumors using ML have been published [12,13,14,15,16,17]. Most studies have used MR-based methods, whereas some implemented 18F-fluorodeoxyglucose (FDG) positron emission tomography (PET)/CT imaging [18,19]. However, CT-based approaches are extremely limited [20,21], and the combination of DECT and ML for differentiating brain tumors has not been reported.

Therefore, in this study, we evaluated the usefulness of ED and Zeff for the quantitative differentiation of BMs, glioblastomas, and PCNSLs in comparison with the apparent diffusion coefficient (ADC), which is a representative and most commonly used quantitative biomarker in MRI for diagnosing brain tumors. Furthermore, we assessed the relationships between DECT parameters and ADCs to reveal what characteristics of brain tumors that they are actually capturing. We performed ML analysis to identify the best models using quantitative images that maximize diagnostic performance.

## 2. Materials and Methods

### 2.1. Study Population

This retrospective study was approved by our Institutional Review Board, which waived the need for informed consent considering the retrospective nature of this study. Consecutive patients with brain tumors and pathological diagnosis of BM, glioblastoma, and PCNSL between January 2017 and March 2023 in Kagoshima University Hospital were included in the study. All participants were Asian and Japanese. All glioblastomas were diagnosed based on an integrated diagnosis combining histology and a glioma-tailored next-generation sequencing panel developed in our institution [22] and fulfilled the World Health Organization classification of 2021 [23]. The exclusion criteria were as follows: lack of preoperative non-contrast-enhanced DECT or contrast-enhanced MRI; lack of any intratumoral contrast enhancement on MRI; and poor image quality for evaluation due to artifacts.

### 2.2. Image Acquisition

All noncontrast-enhanced DECT images were acquired using a 64-detector dual-layer dual-energy CT scanner (IQon spectral CT, Philips Healthcare, Best, The Netherlands). The scan parameters were as follows: tube voltage = 120 kVp, effective tube current-exposure time product with automodulation = 228 mAs, detector-row configuration = 64 × 0.625 mm, rotation time = 0.4 s, pitch = 0.36, and reconstruction slice thickness = 1.0 mm. After the scan, conventional 120-kVp CT (CTconv), ED, and Zeff images were generated by analyzing acquired spectral data in a dedicated workstation (Intellispace Portal, Philips Healthcare). MR images were acquired using a routine brain tumor examination protocol using 3T scanners (MAGNETOM Prisma, Siemens or Achieva, Philips). The scan parameters of DWI were as follows: repetition time = 4600 ms; echo time = 80 ms; acceleration factor = 3; b-values = 0, 1000 s/mm^2^; field of view = 230 × 230 mm^2^; matrix size = 256 × 256; number of slices = 24; slice thickness = 5 mm; slice gap = 1 mm; and acquisition time = 56 s. The scan parameters of DWI were shared between the two scanners. The scan parameters of other MR images are shown in Supplementary Table S1. The CT/MRI systems were inspected every three months by Philips/Siemens service engineers, and underwent daily phantom-based quality control checks performed by the facility’s radiologic technologists.

### 2.3. Image Analysis

For accurate determination of tumor extent, conventional MR images (T2-weighted image, fluid-attenuated inversion recovery, and contrast-enhanced T1-weighted image) were used as the reference standard. As the first step, all acquired DECT images (CTconv, ED and Zeff) were coregistered with the corresponding MR images using Statistical Parametric Mapping (version 12, Functional Imaging Laboratory, UCL Queen Square Institute of Neurology, London, UK). Subsequently, for each lesion, semiautomatic tumor segmentation was performed to delineate the entire contrast-enhancing region of tumors using ITK-SNAP version 4.0.1 (www.itksnap.org, accessed on 28 October 2025) [24], and the volume of interest was placed on each image excluding the areas of cyst, necrosis, macroscopic hemorrhage, and calcification. In patients with multiple discontinuous lesions, all lesions were included for evaluation as far as possible. For the ADC, another region of interest (ROI) (round, 100 mm^2^) was placed on the normal-appearing cerebral white matter at the level of the semioval center, and normalized relative ADC (rADC: ADC of lesion divided by ADC of normal-appearing white matter) was used for evaluation. The segmentation process was performed by two radiologists with 8 and 6 years of experience in radiology, respectively, with blinded clinical and pathological information. The mean, 10th percentile, and 90th percentile of CTconv, ED, Zeff, and rADC within the tumor were extracted using a Python-based in-house program (Python version 3.10.3), and the averaged values of the measurements provided by two observers were used for analysis.

### 2.4. ML Analysis

To emphasize the primary research objective of evaluating the value of DECT parameters as quantitative imaging indicators, we did not use any high-order radiomic features but only included simple first-order statistics (i.e., mean, 10th percentile, and 90th percentile) for ML analysis. AutoGluon, an open-source ML framework, was used to automatically develop and optimize predictive models [25,26]. AutoGluon simplifies the process of model training by implementing an automated pipeline for feature engineering, model selection, hyperparameter tuning, and ensemble learning.

#### 2.4.1. Computational Environment

All analyses were performed on an on-premises Windows 11 workstation using Docker containerization (python:3.10-slim-buster base image). The environment included AutoGluon-Tabular, pandas, scikit-learn, XGBoost, LightGBM, CatBoost, and related machine learning libraries. JupyterLab served as the development interface. AutoGluon training was limited to 600 s (10 min) per model with bagging and stacking disabled (num_bag_folds = 0, num_stack_levels = 0) to prevent overfitting. All random processes used fixed seed (random_state = 42).

#### 2.4.2. Data Splitting Strategy

Patient-level stratified splitting ensured no data leakage between sets, with all lesions from individual patients assigned to single subsets. The dataset was divided into training (60%), validation (20%), and independent test (20%) sets using stratified random sampling with a fixed random seed (random_state = 42) for reproducibility. Class imbalance was addressed through AutoGluon’s automatic weighting.

#### 2.4.3. Feature Selection and Training

Features with poor inter-observer agreement (CTconv90th, Zeff90th; ICC < 0.60) were excluded a priori. Three feature selection methods were compared: LASSO (LassoCV, 3-fold CV), Gini importance (RandomForest, 100 trees), and recursive feature elimination (RFE with LogisticRegression, max_iter = 2000). AutoGluon utilized “best_quality” preset for optimal performance.

#### 2.4.4. Independent Test Evaluation

Area under the receiver operating characteristic curve (AUC), accuracy, precision, recall, and F1-score for classifying between two groups (BMs vs. glioblastomas, BMs vs. PCNSLs, glioblastomas vs. PCNSLs) were analyzed using ML models for each of the following: DECT alone, rADC alone, and DECT + rADC. The AUC was used as the primary evaluation metric to minimize the effects of class imbalance on model performance assessment.

### 2.5. Statistical Analysis

**Figure 1:**
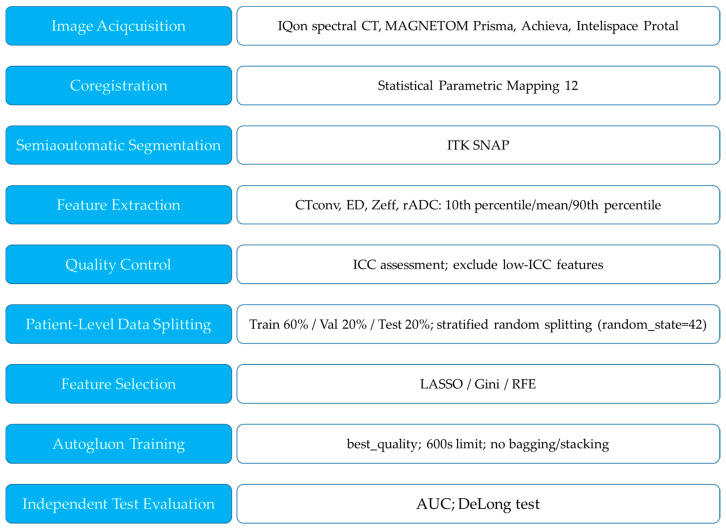
The workflow diagram.

Interobserver agreement was evaluated using the intraclass correlation coefficient (ICC) (ICC = 0.00–0.20, poor correlation; ICC = 0.21–0.40, fair correlation; ICC = 0.41–0.60, moderate correlation; ICC = 0.61–0.80, good correlation; ICC = 0.81–1.00, excellent correlation). Correlations between the mean values of DECT parameters and those of rADC were evaluated using Pearson’s correlation coefficient. The Kruskal–Wallis test was used for between-group comparisons, and post hoc pairwise comparisons were performed using the Mann–Whitney U test with Bonferroni correction. DeLong test was performed to validate AUC differences between ML models. All statistical analyses were performed using MedCalc (version 22.041, MedCalc, Mariakerke, Belgium). *p*-values < 0.05 were used to denote statistical significance. The workflow diagram is shown in Figure 1.

## 3. Results

### 3.1. Patient Characteristics

**Figure 2:**
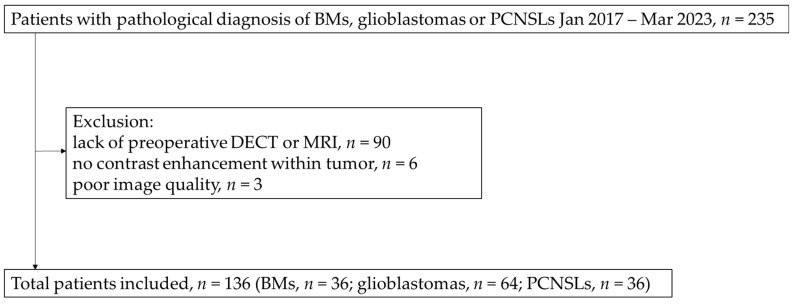
Patient selection flow chart. BMs, brain metastases; PCNSLs, primary central nervous system lymphomas.

In this study, 136 patients (36 BMs, 64 glioblastomas, and 36 PCNSLs) were evaluated (Figure 2). The primary BM lesions were as follows: lung cancer (*n* = 19); breast cancer (*n* = 5); colon cancer (*n* = 2), gastric cancer (*n* = 2), uterine cancer (*n* = 1), bladder cancer (*n* = 1), ureteral cancer (*n* = 1), malignant soft tissue tumor (*n* = 1), skin cancer (*n* = 1), malignant bone tumor (*n* = 1), and unknown (*n* = 2). All patients with PCNSL were histologically classified as having diffuse large B-cell lymphoma. No significant differences in patient sex or age were observed between the three groups (Table 1). The interval between CT and MRI examinations was at most within 2 weeks.

### 3.2. Interobserver Agreement

ICCs were excellent for CTconv10th, CTconvmean, ED10th, EDmean, Zeff10th, Zeffmean, rADC10th, rADCmean, and rADC90th; good for ED90th; and moderate for CTconv90th and Zeff90th.

### 3.3. Data Comparisons

**Figure 3:**
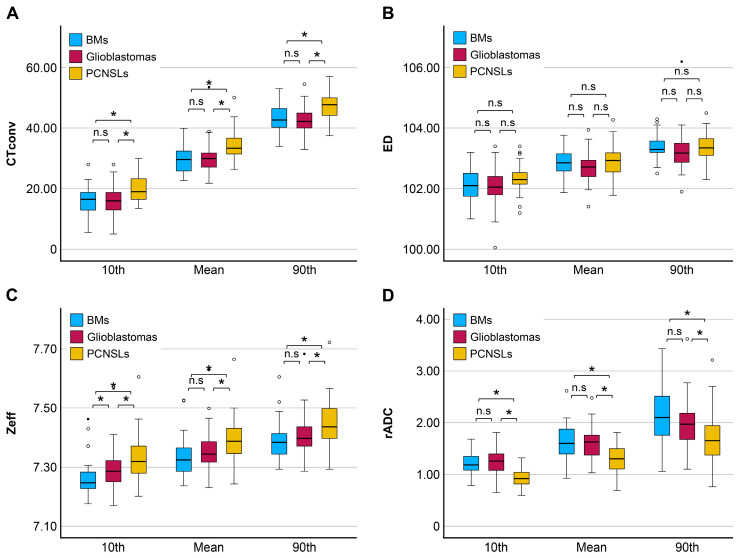
Multiple comparisons of the quantitative values between BMs, glioblastomas, and PCNSLs. Hounsfield units in conventional 120-kVp CT (**A**), electron density (**B**), effective atomic number (**C**), and relative apparent diffusion coefficient (**D**) * *p* < 0.05; n.s, not significant.

Table 2 summarizes the comparison results for the quantitative values among tumor types. In the comparisons of the three groups, all indices of CTconv, Zeff, and rADC exhibited significant differences (*p* < 0.001). In the post hoc pairwise comparisons of BMs versus glioblastomas, only Zeff exhibited a significant difference (10th, *p* = 0.02). Between BMs and PCNSLs, CTconv (10th; mean; 90th, each *p* = 0.002), Zeff (10th; mean; 90th, each *p* < 0.001), and ADC (10th; mean; 90th, each *p* < 0.001) exhibited significant differences. Between glioblastomas and PCNSLs, CTconv (10th; mean; 90th, *p* < 0.001), Zeff (10th; mean; 90th, *p* = 0.01–0.02), and ADC (10th; mean; 90th, *p* < 0.001) exhibited significant differences. Figure 3 presents multiple quantitative value comparison graphs. When analyzed separately according to imaging parameters, PCNSLs exhibited significantly higher values than the other two tumor types in CTconv and significantly lower values in ADC, showing a similar trend. In contrast, Zeff not only exhibited high values for PCNSLs but also demonstrated differences between BMs and glioblastomas, exhibiting a slightly different trend from conventional imaging parameters, such as CTconv and ADC. ED exhibited no significant differences among the three tumor types.

Table 3 presents the AUCs for differentiating each two groups. In the differentiation between BMs and glioblastomas, Zeff exhibited the highest discriminative ability; however, the AUC was 0.66, which was not sufficiently high for diagnosis. In the differentiations involving PCNSLs, CTconv and Zeff exhibited moderate discriminative ability; however, rADC exhibited an even higher AUC.

**Figure 4:**
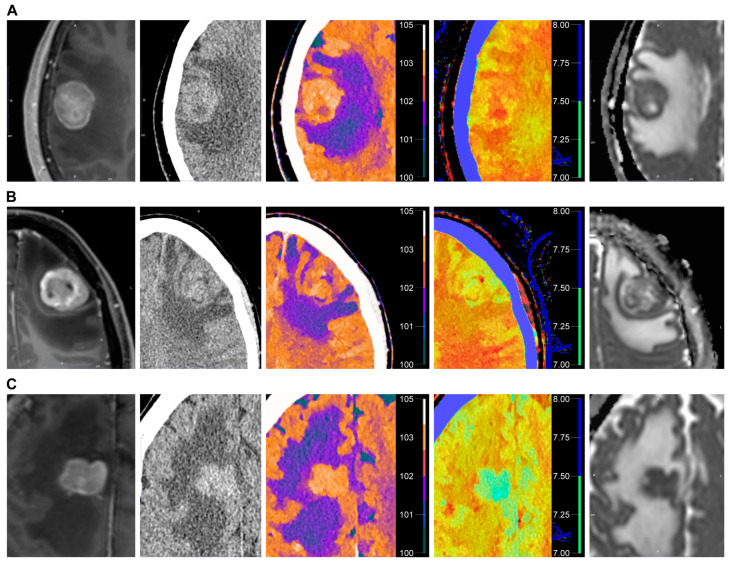
Representative images from a 57-year-old woman with brain metastasis of breast cancer (**A**), a 38-year-old woman with glioblastoma, IDH-wildtype (**B**), and a 74-year-old man with diffuse large B-cell lymphoma (**C**). From left to right, contrast-enhanced T1-weighted image, conventional 120-kVp CT, electron density map, effective atomic number map, and apparent diffusion coefficient map.

Figure 4 presents the DECT and MR images of representative cases of BM, glioblastoma, and PCNSL.

### 3.4. Correlation Analysis

**Figure 5:**
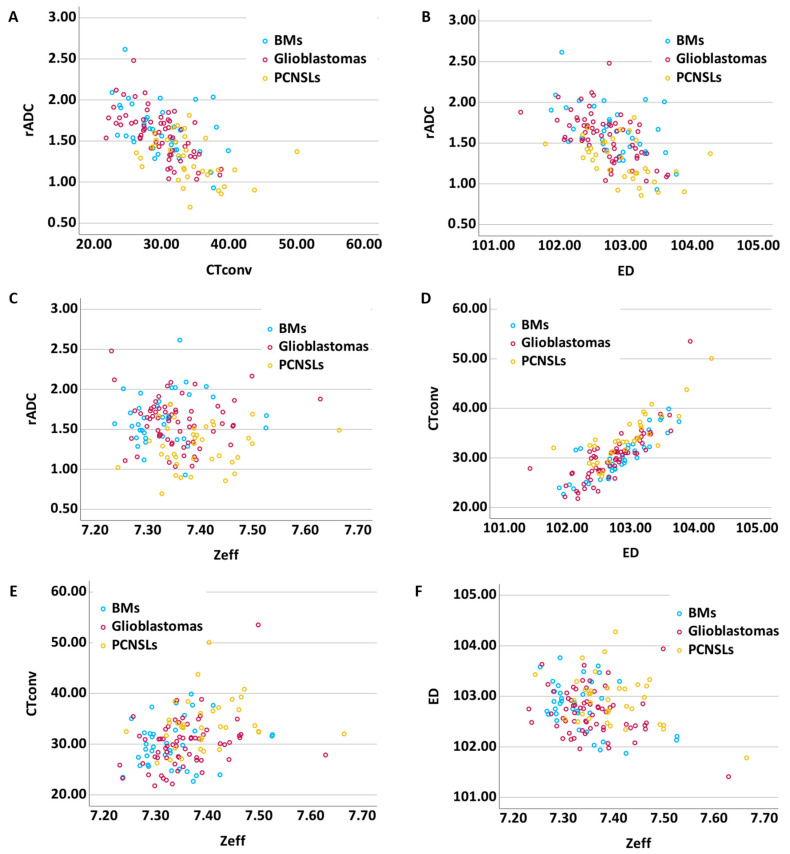
Scatter plots of mean values. CTconv-rADC (**A**), ED-rADC (**B**), Zeff-rADC (**C**), CTconv-ED (**D**), CTconv-Zeff (**E**), and ED-Zeff (**F**). CTconv, conventional 120-kVp CT; ED, electron density; Zeff, effective atomic number; rADC, relative apparent diffusion coefficient.

Figure 5 presents scatter plots of the mean values. CTconv and ED exhibited a moderate negative correlation with rADC (CTconv-ADC, r = −0.48; ED-ADC, r = −0.48). However, Zeff exhibited no correlation with ADC (r = −0.09). Across the DECT parameters, although ED was strongly correlated with CTconv (r = 0.82), only a weak correlation was observed between Zeff and CTconv (r = 0.32). The correlation between ED and Zeff was also weak (r = −0.27).

### 3.5. ML Analysis

**Figure 6:**
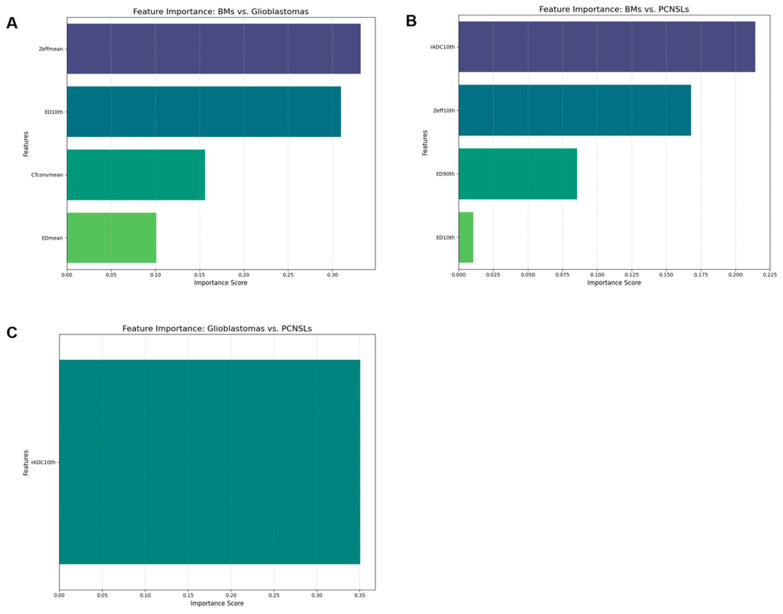
Feature importance for the best models. BMs vs. Glioblastomas (**A**), BMs vs. PCNSLs (**B**), Glioblastomas vs. PCNSLs (**C**). CTconv, conventional 120-kVp CT; ED, electron density; Zeff, effective atomic number; rADC, relative apparent diffusion coefficient; 10th, 10th percentile; 90th, 90th percentile.

Table 4 summarizes the diagnostic performances of the best models for each two-group differentiation in the test set. Feature selection method, selected features, and the diagnostic performances of training and validation sets are summarized in Supplementary Tables S2 and S3. Due to low ICC values, the 90th percentile of CTconv and Zeff were excluded from the analysis. DECT-based ML models exhibited high AUC for all pairwise differentiations (BMs vs. Glioblastomas: AUC = 0.83; BMs vs. PCNSLs: AUC = 0.91; Glioblastomas vs. PCNSLs: AUC = 0.82). Combined models of DECT and rADC demonstrated excellent diagnostic performance between BMs and PCNSLs (AUC = 1) and between Glioblastomas and PCNSLs (AUC = 0.93). In the comparisons of BMs vs. Glioblastomas and BMs vs. PCNSLs, the models that included DECT were identified as the best models, and feature importance analysis revealed that indices related to Zeff and ED were among the most important features (Figure 6). No significant differences in AUC were observed between models in any of the pairwise comparisons.

## 4. Discussion

In this study, rADC was used as a comparator for DECT. The reason for choosing rADC was not only because it is the most commonly used and representative quantitative MRI biomarker but also because numerous studies have reported that ED and Zeff in tumors are associated with cellular density and the nucleus-to-cytoplasmic (N:C) ratio [27,28,29,30,31,32,33]. By comparing ADC, which reflects these histological structures, with ED and Zeff, we attempted to clarify what the new imaging parameters represent in brain tumors. PCNSLs tend to demonstrate a lower ADC value than BMs and glioblastomas [34,35]. This is believed to be due to the extremely high cellular density and N:C ratio in PCNSLs. The Hounsfield unit on conventional CT was reportedly higher in PCNSLs than in BMs or glioblastomas for the same reason [36]. In contrast, unfortunately, these quantitative biomarkers are useless in differentiating BMs from glioblastomas [37]. The comparisons in our cohort exhibited results consistent with those of previous studies. Some studies on cerebral gliomas have suggested a correlation between DECT-derived ED and histological structures, such as cellular density and N:C ratio [27,28]. In those studies, ED was significantly higher in high-grade gliomas than in low-grade gliomas, which is believed to be due to the increased cellular density and N:C ratio in high-grade gliomas. In contrast to previous studies, ED did not yield favorable results in our cohort; however, ED was found to be correlated with CTconv and ADC, supporting the idea that ED likely reflects cellular density and N:C ratio.

To the best of our knowledge, no previous study has evaluated DECT-derived Zeff in brain tumors. Therefore, its diagnostic value is unclear, and what specific tumor characteristics it reflects is it is also unknown. Other than the brain, prior studies have indicated that Zeff of malignant lesions is greater than that of benign lesions in various organs [28,29,30,31,32]. In those reports, higher Zeff values in malignant lesions were hypothetically attributed to higher cellular density and N:C ratio as in the reports on ED. However, significantly higher Zeff values were observed in glioblastomas than in BMs, which is not fully explainable by histological structures such as cellular density and the N:C ratio hypothesized in previous studies. Furthermore, Zeff was not correlated with ADC and exhibited weak correlations with CTconv and ED. From these points, it is unlikely that the variation in Zeff is attributable to histological structures and other inherent tumor characteristics.

Because each DECT parameter alone did not achieve sufficient diagnostic performance, the optimal diagnostic models were explored using ML analysis. With the use of ML, the diagnostic abilities were dramatically improved with DECT in all pairwise comparisons. Particularly for differentiation between BMs and glioblastomas, because a reliable quantitative indicator for differentiating these tumors is currently sparse, the results suggested the potential of DECT as a new promising indicator.

In differentiations involving PCNSLs, combined models of DECT and rADC demonstrated excellent diagnostic performance and DECT is considered useful as a diagnostic tool for differentiations involving PCNSLs as well.

In constructing ML models, simple first-order statistics were used exclusively to prioritize model interpretability and ease of clinical implementation. Although the final AutoGluon model is a weighted ensemble of multiple algorithms, the use of basic features facilitates the understanding of the results by clinicians.

The clinical significance of this study lies in the fact that it was conducted using CT. DECT does not have sufficient diagnostic capability to replace MRI; it remains merely a supplementary tool to MRI. However, brain CT is an essential preoperative examination for brain tumors, and we believe that this work is valuable because it has revealed new diagnostic possibilities from spectral data obtained through routine examinations. With the dual-layer detector DECT we used, spectral data can be obtained in all cases without requiring any special scanning process, which increases its potential for practical use.

Our study has several limitations that should be considered. First, this was a retrospective, single-center study, and the sample sizes were small, particularly for BMs and PCNSLs. There is also a large missing data component due to the absence of DECT examinations. The accuracy of the data has not been sufficiently validated (e.g., observer variation, absence of calibration standards, reproducibility and temporal variations due to clinical status and disease progression). A multicenter prospective study with a larger sample size is necessary to validate our results. Furthermore, clinical information such as age and sex was not taken into account in our machine learning model. A multimodal scoring approach which integrates imaging features and clinical data may further improve diagnostic performance [38]. Second, ADC data acquired from two MRI scanners were used. To overcome this problem, the ADC values were normalized by adding another ROI on the normal-appearing white matter and calculating the relative ADC value. Third, the entire tumor was not fully evaluated in this study because only the contrast-enhancing region of the tumors was included. In particular, glioblastomas and PCNSLs are expected to have non-enhancing regions to some extent; however, accurately delineating non-enhancing tumor boundaries on imaging is difficult. A truly comprehensive evaluation of the entire tumor may provide new insights. Finally, the relationship between the DECT images and the characteristics of the corresponding resected specimens was not evaluated. The integration of DECT images with pathological and genomic information is necessary to elucidate the biological importance of novel imaging biomarkers.

## 5. Conclusions

DECT-derived Zeff differed between BMs, glioblastomas, and PCNSLs. Furthermore, the ML model using DECT parameters achieved high differentiating abilities. This study showed the potential of DECT-derived ED and Zeff as novel quantitative imaging biomarkers for differentiating BMs, glioblastomas, and PCNSLs. Although DECT alone cannot be considered sufficient, it may serve as a useful aid in differential diagnosis.

## Figures and Tables

**Table 1:** Patient characteristics.

Characteristics	BMs (*n* = 36)	Glioblastomas (*n* = 64)	PCNSLs (*n* = 36)	*p*-Value
Age (y)	65 ± 12	69 ± 14	70 ± 13	0.09
Sex				0.61
Male	26	40	24	
Female	10	24	12	
Solitary/multiple				0.08
Solitary	25	51	21	
Multiple	11	13	15	
Primary site (metastases)				
Lung	19	N/A	N/A	
Breast	5	N/A	N/A	
Colon	2	N/A	N/A	
Stomach	2	N/A	N/A	
Uterus	1	N/A	N/A	
Bladder	1	N/A	N/A	
Ureter	1	N/A	N/A	
Soft tissue	1	N/A	N/A	
Skin	1	N/A	N/A	
Bone	1	N/A	N/A	
Unknown	2	N/A	N/A	
IDH-status (glioblastoma)				
Wild type	N/A	64	N/A	
Subtype (PCNSL)				
DLBCL	N/A	N/A	36	

Ages are presented as means ± standard deviations. BMs, brain metastases; PCNSL, primary central nervous system lymphoma; DLBCL, diffuse large B-cell lymphoma; N/A, Not Applicable.

**Table 2:** Comparison of quantitative values between tumor types.

				*p*-Value			
	BMs	Glioblastomas	PCNSLs	Kruskal–Wallis Test	BMs vs. Glioblastomas	BM vs. PCNSLs	Glioblastomas vs. PCNSLs
**CTconv (HU)**							
** 10th**	15.83 ± 4.79	15.82 ± 4.83	19.93 ± 4.27	<0.001	>0.99	0.002	<0.001
** Mean**	30.01 ± 4.66	29.98 ± 4.90	34.05 ± 4.84	<0.001	>0.99	0.002	<0.001
** 90th**	43.11 ± 4.55	43.09 ± 7.89	47.00 ± 4.63	<0.001	>0.99	0.002	<0.001
**ED (%EDW)**							
** 10th**	102.11 ± 0.56	102.07 ± 0.52	102.37 ± 0.47	0.01	>0.99	0.16	0.07
** Mean**	102.82 ± 0.50	102.70 ± 0.45	102.94 ± 0.50	0.06	0.62	>0.99	0.06
** 90th**	103.38 ± 0.44	103.21 ± 0.57	103.38 ± 0.47	0.06	0.16	>0.99	0.16
**Zeff**							
** 10th**	7.26 ± 0.06	7.29 ± 0.07	7.33 ± 0.08	<0.001	0.02	<0.001	0.02
** Mean**	7.33 ± 0.06	7.36 ± 0.07	7.40 ± 0.07	<0.001	0.08	<0.001	0.01
** 90th**	7.39 ± 0.07	7.42 ± 0.10	7.45 ± 0.08	<0.001	0.26	<0.001	0.02
**rADC**							
** 10th**	1.22 ± 0.21	1.26 ± 0.24	0.93 ± 0.16	<0.001	>0.99	<0.001	<0.001
** Mean**	1.64 ± 0.32	1.60 ± 0.30	1.28 ± 0.27	<0.001	>0.99	<0.001	<0.001
** 90th**	2.14 ± 0.51	1.96 ± 0.43	1.70 ± 0.50	<0.001	0.20	<0.001	<0.001

All *p*-values are Bonferroni-corrected. BMs, brain metastases; PCNSLs, primary central nervous system lymphomas; CTconv, conventional 120-kVp CT; ED, electron density; Zeff, effective atomic number; rADC, relative apparent diffusion coefficient; 10th, 10th percentile of data; 90th, 90th percentile of data.

**Table 3:** Comparison of AUCs for differentiating between tumor types.

	AUC (95% CI)		
Parameter	BMs vs. Glioblastomas	BMs vs. PCNSLs	Glioblastomas vs. PCNSLs
CTconv			
10th	0.49 (0.39–0.59)	0.73 (0.61–0.83)	0.73 (0.63–0.82)
Mean	0.51 (0.41–0.62)	0.74 (0.62–0.83)	0.76 (0.66–0.84)
90th	0.54 (0.44–0.64)	0.73 (0.61–0.83)	0.76 (0.67–0.84)
Zeff			
10th	0.66 (0.56–0.76)	0.79 (0.68–0.88)	0.66 (0.56–0.75)
Mean	0.63 (0.53–0.73)	0.77 (0.66–0.86)	0.68 (0.58–0.77)
90th	0.60 (0.50–0.70)	0.75 (0.63–0.84)	0.67 (0.57–0.76)
rADC			
10th	0.55 (0.45–0.65)	0.88 (0.78–0.94)	0.88 (0.80–0.94)
Mean	0.53 (0.43–0.63)	0.81 (0.69–0.89)	0.78 (0.69–0.86)
90th	0.61 (0.51–0.71)	0.74 (0.63–0.84)	0.68 (0.57–0.77)

AUC, area under the receiver operating characteristic curve; 95% CI, 95% confidence interval; CTconv, conventional 120-kVp CT; Zeff, effective atomic number; rADC, relative apparent diffusion coefficient; BMs, brain metastases; PCNSLs, primary central nervous system lymphomas; 10th, 10th percentile of data; 90th, 90th percentile of data.

**Table 4:** Diagnostic performance of the machine learning model for each differentiation in the test set.

		Test Set				
	Model	Accuracy	Precision	Recall	F1	AUC
BMs vs. Glioblastomas						
DECT	Weighted ensemble	0.65	0.5	1	0.67	0.83
rADC	Weighted ensemble	0.65	0	0	0	0.62
DECT + rADC	Weighted ensemble	0.65	0	0	0	0.76
BM vs. PCNSLs						
DECT	Weighted ensemble	0.83	0.86	0.86	0.86	0.91
rADC	Weighted ensemble	0.92	1	0.88	0.93	1
DECT + rADC	Weighted ensemble	1	1	1	1	1
Glioblastomas vs. PCNSLs						
DECT	Weighted ensemble	0.68	0.85	0.73	0.79	0.82
rADC	Weighted ensemble	0.84	1	0.8	0.89	0.94
DECT + rADC	Weighted ensemble	0.74	1	0.67	0.8	0.93

AUC, area under the receiver operating characteristic curve; DECT, dual-energy CT; rADC, relative apparent diffusion coefficient; BMs, brain metastases; PCNSLs, primary central nervous system lymphomas.

## Data Availability

The original data presented in the study are openly available in FigShare at [https://doi.org/10.6084/m9.figshare.30280543].

## Supplementary Materials

The following supporting information can be downloaded at: https://www.mdpi.com/article/10.3390/tomography11110120/s1, Table S1: Scan parameters of the MR images (T2WI, FLAIR, CE-T1WI); Table S2: Feature selection method and selected features in the machine learning models for each differentiation; Table S3: Diagnostic performance of the machine learning models for each differentiation in the training and validation sets.

## Abbreviations

The following abbreviations are used in this manuscript:

ADC	Apparent diffusion coefficient
rADC	Relative apparent diffusion coefficient
AUC	Area under the receiver operating characteristic curve
BM	Brain metastasis
CTconv	Conventional 120-kVp CT
DECT	Dual-energy CT
DWI	Diffusion weighted imaging
ED	Electron density
ICC	Intraclass correlation coefficient
Lasso	Least absolute shrinkage and selection operator
ML	Machine learning
N:C ratio	Nucleus-to-cytoplasmic ratio
PCNSL	Primary central nervous system lymphoma
RFE	Recursive feature elimination
ROI	Region of interest
Zeff	Effective atomic number

## References

[1] Miller K.D., Ostrom Q.T., Kruchko C., Patil N., Tihan T., Cioffi G., Fuchs H.E., Waite K.A., Jemal A., Siegel R.L. (2021). Brain and other central nervous system tumor statistics, 2021. CA Cancer J. Clin..

[2] Fox B.D., Cheung V.J., Patel A.J., Suki D., Rao G. (2011). Epidemiology of metastatic brain tumors. Neurosurg. Clin. N. Am..

[3] Hung N.D., Anh N.N., Minh N.D., Huen D.K., Duc N.M. (2023). Differentiation of glioblastoma and primary central nervous system lymphomas using multiparametric diffusion and perfusion magnetic resonance imaging. Biomed. Rep..

[4] Yamasaki F., Takayasu T., Nosaka R., Amatya V.J., Doskaliyev A., Akiyama Y., Tominaga A., Takeshima Y., Sugiyama K., Kurisu K. (2015). Magnetic resonance spectroscopy detection of high lipid levels in intraaxial tumors without central necrosis: A characteristic of malignant lymphoma. J. Neurosurg..

[5] Hünemohr N., Paganetti H., Greilich S., Jäkel O., Seco J. (2014). Tissue decomposition from dual energy CT data for MC based dose calculation in particle therapy. Med. Phys..

[6] Hua C., Shapira N., Merchant T.E., Klahr P., Yagil Y. (2018). Accuracy of electron density, effective atomic number, and iodine concentration determination with a dual-layer dual-energy computed tomography system. Med. Phys..

[7] Li M., Zheng X., Li J., Yang Y., Lu C., Xu H., Yu B., Xiao L., Zhang G., Hua Y. (2012). Dual-energy computed tomography imaging of thyroid nodule specimens. Investig. Radiol..

[8] Pérez V.G., Arana E., Barrios M., Bartrés A., Cruz J., Montero R., González M., Deltoro C., Martínez-Pérez E., De Aguiar-Quevedo K. (2016). Differentiation of benign and malignant lung lesions: Dual-energy computed tomography findings. Eur. J. Radiol..

[9] Jia Y., Xiao X., Sun Q., Jiang H.C.T. (2018). spectral parameters and serum tumour markers to differentiate histological types of cancer histology. Clin. Radiol..

[10] Kim C., Kim W., Park S.J., Lee Y.H., Hwang S.H., Yong H.S., Oh Y.-W., Kang E.-Y., Lee K.Y. (2020). Application of dual-energy spectral computed tomography to thoracic oncology imaging. Korean J. Radiol..

[11] Lv Y., Zhou J., Lv X., Tian L., He H., Liu Z., Wu Y., Han L., Sun M., Yang Y. (2020). Dual-energy spectral CT quantitative parameters for the differentiation of Glioma recurrence from treatment-related changes: A preliminary study. BMC Med. Imaging.

[12] Zhang Y., Zhang H., Zhang H., Ouyang Y., Su R., Yang W., Huang B. (2024). Glioblastoma and Solitary Brain Metastasis: Differentiation by integrating demographic-MRI and deep-learning radiomics signatures. J. Magn. Reson. Imaging.

[13] Swinburne N.C., Schefflein J., Sakai Y., Oermann E.K., Titano J.J., Chen I., Tadayon S., Aggarwal A., Doshi A., Nael K. (2019). Machine learning for semi¬automated classification of glioblastoma, brain metastasis and central nervous system lymphoma using magnetic resonance advanced imaging. Ann. Transl. Med..

[14] Priya S., Liu Y., Ward C., Le N.H., Soni N., Maheshwarappa R.P., Monga V., Zhang H., Sonka M., Bathla G. (2021). Machine learning based differentiation of glioblastoma from brain metastasis using MRI derived radiomics. Sci. Rep..

[15] Tariciotti L., Caccavella V.M., Fiore G., Schisano L., Garrabba G., Borsa S., Giordano M., Palmisciano P., Remoli G., Remore L.G. (2022). A deep learning model for preoperative differentiation of glioblastoma, brain metastasis and primary central nervous system lymphoma: A pilot study. Front. Oncol..

[16] Liu Y., Li T., Fan Z., Li Y., Sun Z., Li S., Liang Y., Zhou C., Zhu Q., Zhang H. (2022). Image-based differentiation of intracranial metastasis from glioblastoma using automated machine learning. Front. Neurosci..

[17] Zhu F.Y., Sun Y.F., Yin X.P., Zhang Y., Xing L.H., Ma Z.P., Xue L.-Y., Wang J.-N. (2023). Using machine learning-based radiomics to differentiate between glioma and solitary brain metastasis from lung cancer and its subtypes. Discov. Onc..

[18] Jekel L., Brim W.R., Reppert M.V., Staib L., Petersen G.C., Merkai S., Subramanian H., Zeevi T., Payabvash S., Bousabarah K. (2022). Machine learning applications for differentiation of glioma from brain metastasis—A systematic review. Cancers.

[19] Petersen G.C., Shatalov J., Verma T., Brim W.R., Subramanian H., Brackett A., Bahar R., Merkaj S., Zeevi T., Staib L. (2022). Machine Learning in Differentiating Gliomas from Primary CNS Lymphomas: A Systematic Review, Reporting Quality, and Risk of Bias Assessment. AJNR Am. J. Neuroradiol..

[20] Mărginean L., Ștefan P.A., Lebovici A., Opincariu I., Csutak C., Lupean R.A., Coroian P.A., Suciu B.A. (2022). CT in the differentiation of gliomas from brain metastases: The radiomics analysis of the peritumoral zone. Brain Sci..

[21] Lu G., Zhang Y., Wang W., Miao L., Mou W. (2022). Machine learning and deep learning ct-based models for predicting the primary central nervous system lymphoma and glioma types: A multicenter retrospective study. Front. Neurol..

[22] Higa N., Akahane T., Yokoyama S., Yonezawa H., Uchida H., Takajo T., Kirishima M., Hamada T., Matsuo K., Fujio S. (2020). A tailored next-generation sequencing panel identified distinct subtypes of wildtype IDH and TERT promoter glioblastomas. Cancer Sci..

[23] Louis D.N., Perry A., Wesseling P., Bart D.J., Cree I.A., Figarella-Branger D., Hawkins C., Ng H.K., Pfister S.M., Reifenberger G. (2021). The 2021 WHO classification of tumors of the central nervous system: A summary. Neuro-Oncology.

[24] Yushkevich P.A., Piven J., Hazlett H.C., Smith R.G., Ho S., Gee J.C., Gerig G. (2006). User-guided 3D active contour segmentation of anatomical structures: Significantly improved efficiency and reliability. NeuroImage.

[25] Erickson N., Mueller J., Shirkov A., Zhang H., Larroy P., Li M., Smola A. (2020). AutoGluon-Tabular: Robust and Accurate AutoML for Structured Data. arXiv.

[26] Autogluon. https://auto.gluon.ai/.

[27] Kaichi Y., Tatsugami F., Nakamura Y., Baba Y., Iida M., Higaki T., Kiguchi M., Tsushima S., Yamasaki F., Amatya V.J. (2018). Improved differentiation between high- and low-grade gliomas by combining dual-energy CT analysis and perfusion CT. Medicine.

[28] Chakrabarti R., Gupta V., Vyas S., Gupta K., Singh V. (2022). Correlation of dual energy computed tomography electron density measurements with cerebral glioma grade. Neuroradiol. J..

[29] Wang P., Xiao Z., Tang Z., Wang J. (2020). Dual-energy CT in the differentiation of stage T1 nasopharyngeal carcinoma and lymphoid hyperplasia. Eur. J. Radiol..

[30] Luo S., Sha Y., Wu J., Lin N., Pan Y., Zhang F., Huang W. (2022). Differentiation of malignant from benign orbital tumours using dual-energy CT. Clin. Radiol..

[31] Wang N., Ju Y., Wu J., Liu A., Chen A., Liu J., Liu Y., Li J. (2019). Differentiation of liver abscess from liver metastasis using dual-energy spectral CT quantitative parameters. Eur. J. Radiol..

[32] Jiang L., Liu D., Long L., Chen J., Lan X., Zhang J. (2022). Dual-source dual-energy computed tomography-derived quantitative parameters combined with machine learning for the differential diagnosis of benign and malignant thyroid nodules. Quant. Imaging Med. Surg..

[33] Mileto A., Allen B.C., Pietryga J.A., Farjat A.E., Zarzour J.G., Bellini L., Ebner L., Morgan D.E. (2017). Characterization of incidental renal mass with dual-energy CT: Diagnostic accuracy of effective atomic number maps for discriminating nonenhancing cysts from enhancing masses. Am. J. Roentgenol..

[34] Lin X., Lee M., Buck O., Woo K.M., Zhang Z., Hatzoglou V., Omuro A., Arevalo-Perez J., Thomas A., Huse J. (2017). Diagnostic accuracy of T1-weighted dynamic contrast-enhanced–MRI and DWI-ADC for differentiation of glioblastoma and primary CNS lymphoma. Am. J. Neuroradiol..

[35] Matuszewska M.N., Bladowska J., Sąsiadek M., Zimmy A. (2018). Differentiation of glioblastoma multiforme, metastases and primary central nervous system lymphomas using multiparametric perfusion and diffusion MR imaging of a tumor core and a peritumoral zone—Searching for a practical approach. PLoS ONE.

[36] Slone H.W., Blake J.J., Shah R., Guttikonda S., Bourekas E.C. (2005). CT and MRI findings of intracranial lymphoma. Am. J. Roentgenol..

[37] Fordham A.J., Hacherl C.C., Patel N., Jones K., Myers B., Abraham M., Gendreau J. (2021). Differentiating glioblastomas from solitary brain metastases: An update on the current literature of advanced imaging modalities. Cancers.

[38] Yu S., Hao R., Cui J., Yang Y., Wang Z., Zheng H., Li M., Li X., Chen W., Jia W. (2025). Integration of radiomic and deep features to reliably differentiate benign renal lesions from renal cell carcinoma. Eur. J. Radiol..

