# Supplementary material for: Electron Density and Effective Atomic Number as Quantitative Biomarkers for Differentiating Malignant Brain Tumors: An Exploratory Study with Machine Learning
Source: Tomography. 2025 Oct 29;11(11):120. doi: 10.3390/tomography11110120 (PMC12656531; doi:10.3390/tomography11110120)
Supplement: Supplementary file 1 [file tomography-11-00120-s001.zip › tomography-3894831-supplementary.pdf]

Supplementary Materials

Supplementary Table. S1 Scan parameters of the MR images (T2WI, FLAIR, CE-T1WI).

	**T2WI**	**FLAIR**	**CE-T1WI (MPRAGE or 3D-TFE)**
TE (ms)	91	121	1.94 or 3.79
TR (ms)	4000	9000	2000 or 8.14
TI (ms)	N/A	2530	900 or 1044
Acceleration factor	2	2	2 or 3
FOV (mm^2)	230 × 230	230 × 230	230 × 230 or 240 × 240
Matrix size	512 × 512	512 × 512	491 × 491 or 480 × 480
Number of slices	24	24	80
Slice thickness (mm)	5	5	2
Slice gap (mm)	1	1	N/A
Acquisition time (s)	80	126	253 or 170

T2WI, T2-weighted imaging; FLAIR, fluid-attenuated inversion recovery imaging; CE-T1WI, contrast-enhanced T1-weighted imaging; MPRAGE, magnetization-prepared rapid gradient echo; 3D-TEF, three-dimensional turbo field echo; TE, echo time; TR, repetition time; TI, inversion time; FOV, field of view; N/A, not applicable.

Supplementary Table. S2 Feature selection method and selected features in the machine learning models for each differentiation.

	Feature selection method	Selected features
BMs vs. Glioblastomas		
DECT	Gini	CT_{conv} (mean), ED (10th, mean), Z_{eff} (mean)
rADC	Lasso	rADC (10th, 90th)
DECT + rADC	Gini	ED (10th, mean), Z_{eff} (mean), rADC (90th)
BMs vs. PCNSLs		
DECT	Lasso	CT_{conv} (10th, mean), ED (10th, mean, 90th), Z_{eff} (10th, mean)
ADC	RFE	rADC (10th)
DECT + rADC	Lasso	ED (10th, 90th), Z_{eff} (10th), rADC (10th)
Glioblastomas vs. PCNSLs		
DECT	Gini	CT_{conv} (10th, mean)
rADC	RFE	rADC (10th)
DECT + rADC	Gini	CT_{conv} (mean), rADC (10th, mean)

DECT, dual-energy CT; CT_{conv}, conventional 120-kVp CT; ED, electron density; Z_{eff}, effective atomic number; rADC, relative apparent diffusion coefficient; BMs, brain metastases; PCNSL, primary central nervous system lymphomas; 10th, 10th percentile of data; 90th, 90th percentile of data

Supplementary Table. S3 Diagnostic performance of the machine learning models for each differentiation in the training and validation sets

		Training set					Validation set				
	Model	Accuracy	Precision	Recall	F1	AUC	Accuracy	Precision	Recall	F1	AUC
BMs *vs.* Glioblastomas											
DECT	Weighted Ensemble	0.88	0.74	0.94	0.83	0.94	0.85	0.75	0.86	0.8	0.98
rADC	Weighted Ensemble	0.81	0.89	0.44	0.59	0.91	0.8	1	0.43	0.6	0.91
DECT + rADC	Weighted Ensemble	0.72	0.63	0.23	0.38	0.77	0.6	0	0	0	0.82
BM *vs.* PCNSLs											
DECT	Weighted Ensemble	0.85	1	0.67	0.8	0.88	0.93	1	0.88	0.93	0.93
rADC	Weighted Ensemble	0.85	0.93	0.72	0.81	0.9	0.87	1	0.75	0.86	0.96
DECT + rADC	Weighted Ensemble	0.98	0.95	1	0.97	1	1	1	1	1	1
Glioblastomas *vs.* PCNSLs											
DECT	Weighted Ensemble	0.79	0.86	0.8	0.83	0.82	0.65	0.75	0.69	0.72	0.63
rADC	Weighted Ensemble	0.84	0.86	0.9	0.88	0.92	0.85	0.86	0.92	0.89	0.89
DECT + rADC	Weighted Ensemble	0.89	0.92	0.9	0.91	0.97	0.8	0.91	0.77	0.83	0.93

DECT, dual-energy CT; rADC, relative apparent diffusion coefficient; BM, brain metastases; PCNSLs, primary central nervous system lymphomas
